# Risk of Secondary Bacterial Infections Revealed by Changes in *Trachinotus ovatus* Skin and Gill Microbiota During a *Cryptocaryon irritans* Infection Cycle

**DOI:** 10.3390/microorganisms13071660

**Published:** 2025-07-14

**Authors:** Naiqi Liang, Li Zhu, Shifeng Wang, Weihao Zhang, Xinlei Lin, Yongcan Zhou, Haizhu Ke, Shanheng Yuan, Meijing Li, Yan Cai

**Affiliations:** 1 Hainan Provincial Key Laboratory for Tropical Hydrobiology and Biotechnology, School of Marine Biology and Fisheries, Hainan University, Haikou 570228, China; 15351852578@163.com (N.L.); 18891443097@163.com (L.Z.); 17693186286@163.com (W.Z.); lxleipr@163.com (X.L.); zychnu@163.com (Y.Z.); 15798955883@163.com (H.K.); 13602899421@163.com (S.Y.); 18389510079@163.com (M.L.); 2 Collaborative Innovation Center of Marine Science and Technology, Hainan University, Haikou 570228, China

**Keywords:** *Trachinotus ovatus*, *Cryptocaryon irritans*, microbiota, skin and gill, secondary bacterial infections, safety

## Abstract

This study aims to investigate the response of surface bacterial communities in *Trachinotus ovatus* to *Cryptocaryon irritans* infection at different stages of a single infection cycle (0~168 h). These samples were analyzed using high-throughput 16S rRNA sequencing. Alpha diversity analysis showed a reduction in the richness and diversity of skin microbiota during infection, with partial recovery post-detachment. Beta diversity analysis revealed distinct structural shifts in skin microbiota at early (24 h) and post-detachment (168 h) stages compared to other phases, while gill microbiota remained stable except during detachment. At the phylum level, Proteobacteria, Actinobacteriota, Bacteroidetes, and Firmicutes were dominant on the skin at different stages, whereas the gill microbiota was predominantly Proteobacteria (>90%). At the genus level, opportunistic pathogens, such as *Vibrio* and *Nautella*, increased in relative abundance on the skin with the infection progression, while gill microbiota composition barely changed. The hepatic bacterial load continued to increase with infection duration. These findings indicate that *C. irritans* alters microbiota composition on skin, facilitating pathogen invasion, thereby elevating the risk of secondary bacterial infections in *T. ovatus*.

## 1. Introduction

*Trachinotus ovatus*, commonly known as the golden pompano, is widely distributed globally, spanning the eastern Atlantic, the Mediterranean, and the Asia-Pacific regions. This valuable marine species is extensively farmed in the South China Sea region [1] and is one of the most economically valuable aquaculture species in Asia [1,2]. In the farming of *T. ovatus*, *Cryptocaryon irritans* disease is recognized as the “most devastating parasitic disease” [3,4]. *C. irritans*, also known as “marine ich,” belongs to the ciliate subclass Oligohymenophorea within the family Cryptocaryonidae [5,6]. As a typical marine parasite, it infects most marine teleost fish species. Due to its rapid reproduction and spread, it can lead to up to 100% mortality in *T. ovatus* if not controlled promptly, causing severe economic losses in the marine aquaculture industry [7]. Generally, a single infection cycle lasts approximately 3 to 5 days (120 h) from the onset of infection until the parasite detaches from the host fish. Following infection with *C. irritans*, *T. ovatus* exhibits a series of typical clinical signs and pathological changes. Notably, distinct white spots develop on the skin, gills, and fins, which are caused by vesicles formed as the trophont stage of the parasite embeds into the epidermis [6]. Concurrently, the infected fish may have ulcers on the body surface, fin rot, scale loss, and excessive mucus in the gills. These pathological alterations may ultimately result in the death of *T. ovatus* [3]. Currently, there are limited physical and chemical methods to prevent *C. irritans* infections in fish. Physical methods primarily encompass rotational farming, freshwater or low-salinity immersion, ultraviolet (UV) irradiation, and ozone treatment. Chemical approaches include formalin, copper-based formulations, and traditional Chinese herbal medicine treatments [7]. However, all of these methods have certain limitations, such as potential toxicity to fish or inconsistent effectiveness [8].

In recent years, research on interactions between parasites and microorganisms has provided new avenues for treating parasitic diseases. Interactions between parasites, hosts, and host-associated microbiota are considered essential determinants in disease progression and development. Currently, research on *C. irritans* disease focuses mainly on interactions between the parasite (*C. irritans*) and the host (fish), while overlooking the microbial communities that adhere to the mucosal layers of fish skin and gill tissues [9,10]. Recent studies indicate that host-associated mucosal symbiotic bacteria play an important role in the onset and development of parasitic diseases. Cooperative microbial interactions may promote infection and support the progression of these parasitic diseases [11,12]. Although these protozoan infections are often associated with imbalances or dysbiosis in the microbiota, this observation challenges the single-pathogen origin theory for these parasitic diseases [9,13]. To accurately understand these diseases under natural conditions, it is essential to consider broader relationships (host–parasite–microbiota) rather than just host–parasite interactions. On the other hand, in healthy fish, the internal organs, such as the liver, are near-sterile [14]. However, following infection, bacteria often invade these organs, particularly the liver [15]. It is widely recognized that the severity of bacterial infections in fish is positively correlated with hepatic bacterial load [16].

In this study, we aimed to investigate changes in the symbiotic microbial communities on the skin and gill of *T. ovatus* at various time points during *C. irritans* infection, with the goal of evaluating the association between *C. irritans* and the surface-associated microbiota of *T. ovatus* and exploring the causes of secondary bacterial infections, filling the research gap related to the interaction mechanism between parasitic infections and the surface microbiome of fish.

## 2. Materials and Methods

### 2.1. Fish, C. irritans, and Infection Experiment

#### 2.1.1. Fish

Healthy *T. ovatus* was purchased from Hainan Baotong Yili Aquatic Products Co., Ltd., Haikou, China, with an average weight of 87.1 ± 5.3 g. These fish were fed commercial feed (Guangdong Yuehai Feed Co., Ltd., Zhanjiang, China) at a rate of 3% of the fish’s body weight daily and incubated in PVC barrels (500 L), maintaining a water temperature of 29 ± 1 °C, pH value of 7.8 ± 0.2, salinity of 30 ± 0.5, and dissolved oxygen (DO) of 6.8 ± 0.1 milligrams per liter. Before the experiment, 6 individuals of *T. ovatus* were dissected to collect their gill tissues and body surface mucus. These samples were then observed under a microscope to check for the presence of trophonts, thereby determining whether the fish were infected with *C. irritans* [17].

#### 2.1.2. *C. irritans* Propagation and Collection

The strain of *C. irritans* used was sourced from our laboratory pre-culture (isolated from *T. ovatus* infected with *C. irritans* in an aquaculture base in Lingao, Hainan Province, China) and propagated and collected following the method of [18,19], establishing a stable propagation system to ensure a sufficient number of *C. irritans* larvae for experimental use. First, the *C. irritans* cysts were repeatedly washed in filtered and sterilized seawater to remove surface impurities and then collected in a 24-well plate and placed in a biochemical light incubator for hatching (temperature set at 27 ± 0.5 °C). During the hatching process, the filtered and sterilized seawater was replaced every 24 h. After 60 h of hatching, the larvae were collected and used to infect healthy *T. ovatus* at a dose of 8000 larvae per fish [20]. Once the “white spots” appeared on the body surface of the *T. ovatus*, they were transferred to a 300 L funnel-shaped PVC collection bucket for *C. irritans* and fed there, while collecting the cysts that fell off the fish. The collected cysts were repeatedly washed with filtered and sterilized seawater and then stored at 16 °C for subsequent experiments.

#### 2.1.3. *C. irritans* Infection

A total of 100 fish were used for infection experiments and in a clean PVC barrel (500 L) for temporary rearing. Among them, 10 fish were sampled before infection. Subsequently, infection was carried out using theronts of the protozoan *C irritans* that had hatched within the past 2 h, with an infection dose of 4000 parasites per fish. The infection dose was referenced from the literature published by Hongping Chen in 2021 [21] and was verified by preliminary experiments. During infection, the water volume was controlled at 5 L per fish. Two hours after infection, the remaining 90 fish were transferred to clean PVC barrels (500 L) for further cultivation, maintaining a water temperature of 29 ± 1 °C, pH value of 7.8 ± 0.2, salinity of 30 ± 0.5, and dissolved oxygen (DO) of 6.8 ± 0.1 milligrams per liter. In the meantime, the water volume was controlled at 5 L per fish. Following infection, the swimming posture of the experimental fish was continuously observed at 12 h intervals. Fish with abnormal swimming postures (such as lying flat or even swimming with their bellies up) were removed and euthanized. Euthanasia was performed by immersion in buffered MS-222 (≥500 mg/L; ≥30 min) until cessation of opercular movement.

### 2.2. Sample Collection and Processing

Surface (S) and gill (G) bacterial samples were collected at specific time points and named as follows: samples collected before infection were named S0 and G0 groups, samples collected 24 h post-infection were named S24 and G24 groups (early infection), samples collected 72 h post-infection were named S72 and G72 (mid-stage infection), samples collected 120 h post-infection were named S120 and G120 (late-stage infection), and samples collected 168 h post-infection were named S168 and G168 (post-detachment) for sampling. The skin and gill samples from ten fish were collected at each time point, and the samples from two fish were mixed to form one sample, resulting in 5 parallel samples per time point.

The sampling method involved anesthetizing the experimental fish, rinsing the skin with sterile seawater 2–3 times to remove skin debris, and then collecting skin samples using dry sterile cotton swabs, with each pair of fish forming one sample. The skin bacterial samples were collected by repeatedly wiping the central approximately 1 cm^2^ area of the left side of the fish’s body surface, and the tip of the cotton swab was cut off. The gill bacterial samples were obtained by repeatedly wiping the second gill leaf on the left side. The heads of the cotton swab samples were then placed separately into sterile storage tubes, frozen in liquid nitrogen for subsequent experiments.

### 2.3. Liver Bacterial Load Measurement

After the collection of skin and gill samples, the surface of the *T. ovatus* was thoroughly cleansed with 75% ethanol, followed by dissection conducted under stringent sterile conditions. Subsequently, liver tissues (0.1–0.2 g) from each of the 10 fish at each time point were collected, weighed, and placed into a 1.5 mL centrifuge tube containing sterile saline (tissue/saline = 1:9) for the purpose of grinding. After homogenization of the liver tissues from each time point, a serial dilution with a 10-fold gradient was performed. Aliquots of 100 μL from dilutions with concentrations of 10^0^, 10^−1^, and 10^−2^ were uniformly dispensed onto a general seawater medium, which was then incubated at 30 °C for a duration of 24 h. Following the incubation period, all colonies present on the medium were meticulously enumerated, enabling the calculation of the bacterial load per gram of liver tissue, expressed as CFU/g. The entire sampling procedure was executed under sterile conditions within a laminar flow cabinet. A total of 10 fish were sampled and their liver bacterial loads measured at each time point.

### 2.4. Ethical Statement

In this study, the Animal Use and Care Committee of Hainan University permitted the animal experiments in advance. All possible efforts were made to reduce the suffering of the experimental animals. A protocol (including the research question, key design features, and analysis plan) was prepared before the study, and the protocol was registered with the Animal Use and Care Committee of Hainan University.

### 2.5. DNA Extraction and 16S rRNA High-Throughput Sequencing

The extraction of total DNA was conducted employing the DNeasy^®^ PowerSoil^®^ Pro Kit (QIAGEN, Hilden, Germany), followed by the assessment of DNA sample purity and concentration using the NanoDrop2000 spectrophotometer (Thermo Fisher Scientific, Waltham, MA, USA). The evaluation of DNA integrity was executed via 1% agarose gel electrophoresis.

Amplification of the bacterial 16S rDNA gene within skin samples was achieved through the application of two universal bacterial primers, designated as 338F and 806R. The amplification targeted the 16S V3-V4 hypervariable region. The precise sequences of the primers were as follows: the forward primer 338F was 5′-ACTCCTACGGGAGGCAGCAG-3′, and the reverse primer 806R was 5′-GGACTACHVGGGTWTCTAAT-3′. The PCR amplification protocol included the following: initial denaturation at 95 °C for 3 min; 30 cycles of denaturation at 95 °C for 30 s, annealing at 53 °C for 30 s, and extension at 72 °C for 45 s; and a final extension at 72 °C for 10 min. Upon completion of the reaction, the PCR products were preserved at 10 °C pending subsequent analysis. Illumina MiSeq sequencing and processing of the sequencing data were performed by Majorbio Bio-Pharm Technology Co. Ltd. (Shanghai, China)

### 2.6. Bioinformatics and Statistical Analysis

The quantified and homogenized PCR products were analyzed utilizing the Uparse 11.0 platform. Clustering of Operational Taxonomic Units (OTUs) was conducted on valid sequences at a 97% similarity threshold, resulting in the generation of an OTU table. Following this, the RDP Classifier 2.13 was employed to perform taxonomic analysis on the representative sequences of OTUs at the 97% similarity level, aligning them with the Silva and other 16S bacterial databases to obtain taxonomic annotation information at various hierarchical levels. Subsequently, a statistical analysis of the relative abundance of OTUs at the phylum and genus levels was conducted. Additionally, Alpha and Beta diversity indices were utilized to assess species diversity, richness, and differences among samples, thereby illustrating the composition and structural variations within the microbial communities of each batch of samples. A *p*-value less than 0.05 is deemed indicative of a significant difference. Alpha-diversity indices encompass richness indices (Sobs and Chao1) and diversity indices (Shannon and Simpson), which were calculated and visualized using the vegan 2.6-4 and ggplot2 3.4.2 R packages, respectively. In the context of Beta diversity analysis, the Bray–Curtis dissimilarity matrix and Jaccard similarity coefficients were computed using the microeco 0.14.1 version and visualized through ggplot2. Significance was determined using One-way ANOVA. LEfSe analysis (https://cloud.majorbio.com/page/tools/) was conducted to identify significant correlations between bacterial groups and different sample groups, with each sample group consisting of 5 biological replicates to ensure statistical robustness. The analysis of significant differences was executed using SPSS Statistics 26.0 software (IBM, Armonk, NY, USA).

## 3. Results

### 3.1. Hepatic Bacterial Load

**Figure 1:**
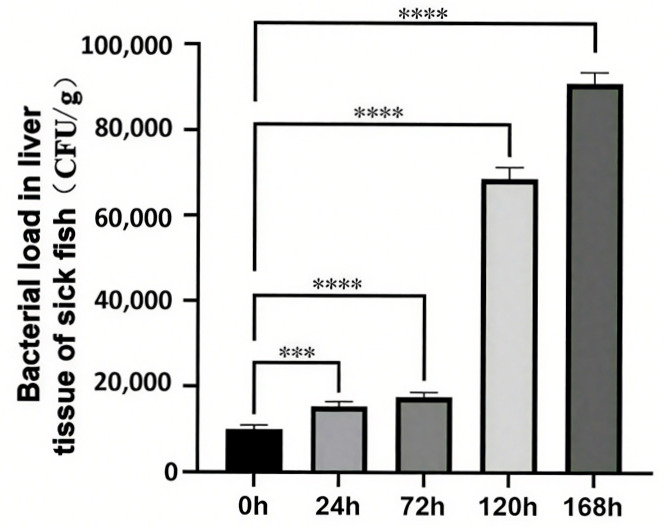
Changes in hepatic bacterial load of *T. ovatus* during *C. irritans* infection. *** represents *p* < 0.001, **** represents *p* < 0.0001.

Following a 24 h exposure to *C. irritans*, a significant increase in bacterial load was observed in the liver. This trend persisted over time, with the bacterial load consistently rising until it reached its peak at 168 h post-infection clearance (Figure 1). Notably, the bacterial load in the uninfected control group (recorded at 0 h) differed markedly from that of all other experimental groups.

### 3.2. Operational Taxonomic Unit (OTU) Analysis

**Figure 2:**
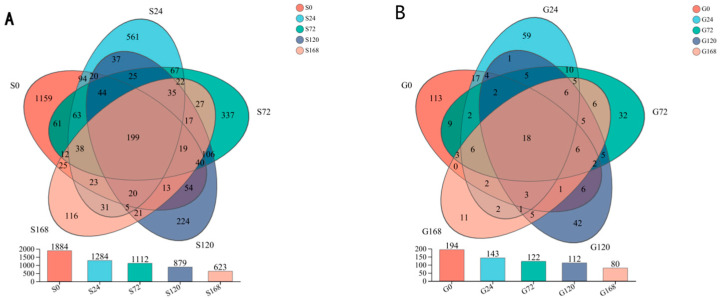
Dynamic changes in the abundance of operational taxonomic units (OTUs) within the microbial community across different time points of *C. irritans* infection. (**A**) Skin (**B**) Gill.

Under conditions of infection at 0 h, 24 h, 72 h, 120 h, and 168 h, the number of core OTUs is 199. The number of unique OTUs at each time point was 1159, 561, 337, 224, and 116, respectively, indicating a clear trend of decreasing unique species with prolonged infection time. This suggests a significant correlation between the duration of infection and the reduction in unique species. A similar decreasing trend was observed in gill OTUs as infection time increased (Figure 2).

### 3.3. Skin and Gill Microbiota Diversity Analysis

#### 3.3.1. Alpha Diversity

**Figure 3:**
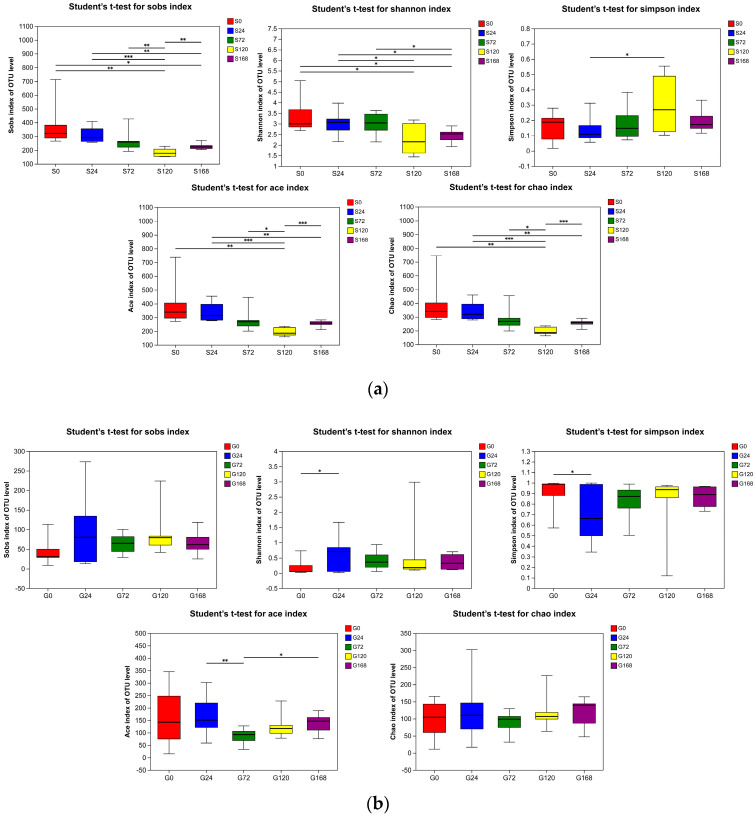
Alpha diversity indices (Sobs, Shannon, Simpson, ACE, and Chao) of bacterial communities in (**a**) Skin and (**b**) Gill. * represents *p* < 0.05, ** represents *p* < 0.001 and *** represents *p* < 0.0001.

In the comparison of skin samples, the S120 group showed a statistically significant decrease in Sobs, Ace, and Chao indices compared to the S0, S24, S72, and S168 groups (*p* < 0.05), indicating a reduced richness of symbiotic skin microbiota at this specific stage. Additionally, the Shannon index in the S120 group was significantly lower than that of the S0 and S24 groups (*p* < 0.05), supporting a notable decline in skin microbiota diversity at this stage (Figure 3a).

In gill samples, changes in alpha diversity indices were minimal. There were no significant differences in Sobs and Chao indices between groups (*p* > 0.05). The G24 group had a significantly higher Shannon index than the G0 group (*p* < 0.05), with no significant differences among other groups (*p* > 0.05). The Simpson index of the G24 group was significantly lower than that of the G0 group (*p* < 0.05), while other groups showed no significant differences (*p* > 0.05). The Ace index in the G72 group was significantly higher than in the G24 and G168 groups (*p* < 0.05), with no significant differences among other groups (*p* > 0.05), suggesting that *C. irritans* infection had no substantial impact on gill microbiota diversity and richness (Figure 3b).

#### 3.3.2. Beta Diversity Analysis

**Figure 4:**
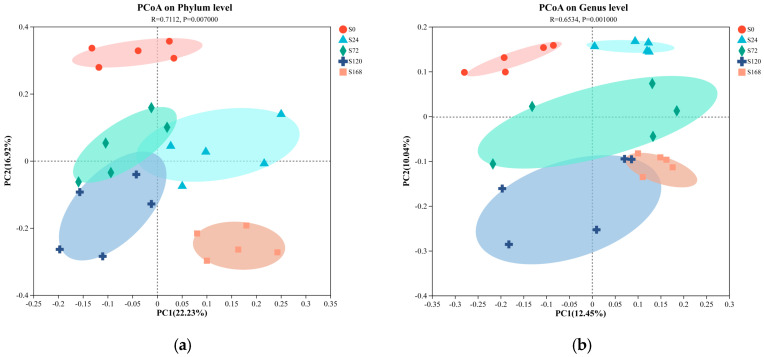

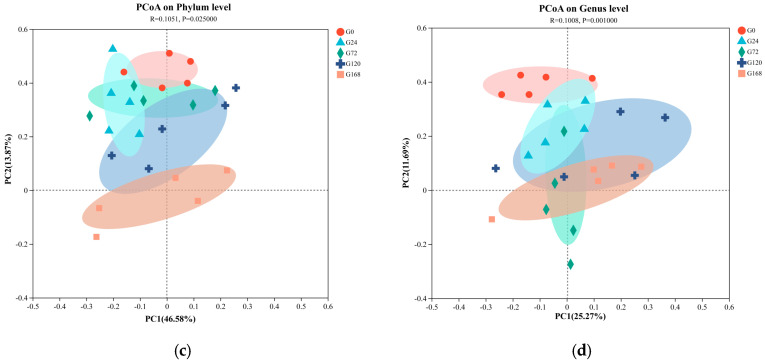
Principal coordinate analysis (PCoA) of microbiome composition at the phylum and genus taxonomic levels, with subplots illustrating the following: (**a**) skin microbiome at the phylum level; (**b**) skin microbiome at the genus level; (**c**) gill microbiome at the phylum level; and (**d**) gill microbiome at the genus level.

Principal Coordinate Analysis (PCoA) based on unweighted UniFrac distances was conducted on fish surface samples. Pairwise ANOSIM tests were also used to detect significant community differences between infection groups. For skin samples, PCoA analysis at the phylum level showed distinct clustering tendencies, with samples from S0 and S168 located at a greater distance from each other, while other groups were closer (Figure 4a). At the genus level, similar to the phylum-level results, the S0 and S168 groups were further apart, indicating significant structural changes in skin microbiota after *C. irritans* infection and further alterations following parasite detachment (Figure 4b).

In gill samples, regardless of phylum or genus levels, strong clustering tendencies were observed, with a minimal distance between groups. Only the G168 group displayed a notable separation from other groups, suggesting that gill microbiota composition was less affected by *C. irritans* infection, with some differences appearing only post-detachment (Figure 4c,d).

### 3.4. Skin and Gill Microbiota Structural Analysis

#### 3.4.1. Phylum Level

**Figure 5:**
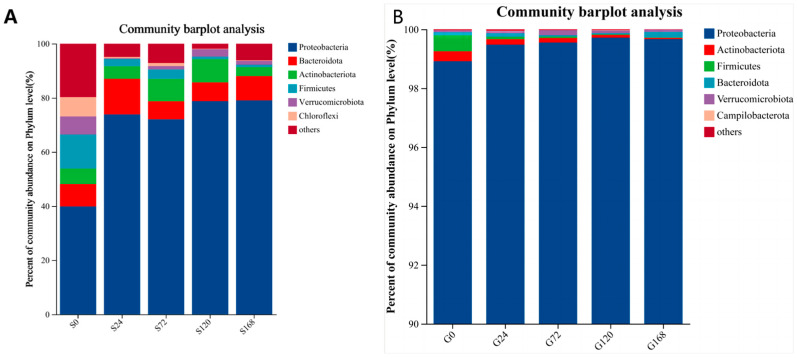
Relative abundance of bacteria at phylum level; (**A**) Skin, (**B**) Gills.

After categorizing skin samples by phylum and ordering by abundance, Proteobacteria consistently held a dominant position, showing a significant increase following *C. irritans* infection and remaining at a stable high level throughout the infection period. In contrast, Actinobacteria abundance decreased significantly at S24 post-infection, from 9.22% to 3.39%. Similarly, Firmicutes abundance dropped from 8.53% to 2.18% at the S24 stage, with a downward trend continuing over time (Figure 5A).

Gill samples categorized by phylum also showed Proteobacteria as the dominant phylum across groups, with an increasing presence of Verrucomicrobiota over time. Firmicutes abundance decreased steadily with infection duration (Figure 5B).

#### 3.4.2. Genus Level

**Figure 6:**
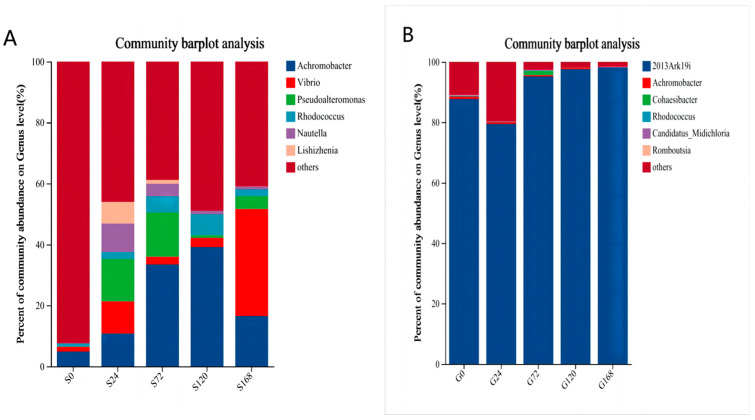
Relative abundance of bacteria at genus level, (**A**) Skin, (**B**) Gills.

In a detailed genus-level synonym of skin samples, *Achromobacter* abundance increased following infection by *C. irritans* (S24 stage). Additionally, Vibrio abundance increased after infection. The study also identified two newly detected genera, *Nautella* and *Pseudoalteromonas*, which peaked in abundance 24 h post-infection and then gradually declined over time (Figure 6A).

Overall, as infection time increased, conditional pathogenic genera abundance in the skin microbiota rose. In gill samples, *2013Ark19i* was consistently the dominant genus, with minor changes in other genera, except during early infection with *C. irritans* (Figure 6B).

#### 3.4.3. LEfSe Multilevel Species Differential Analysis

**Figure 7:**
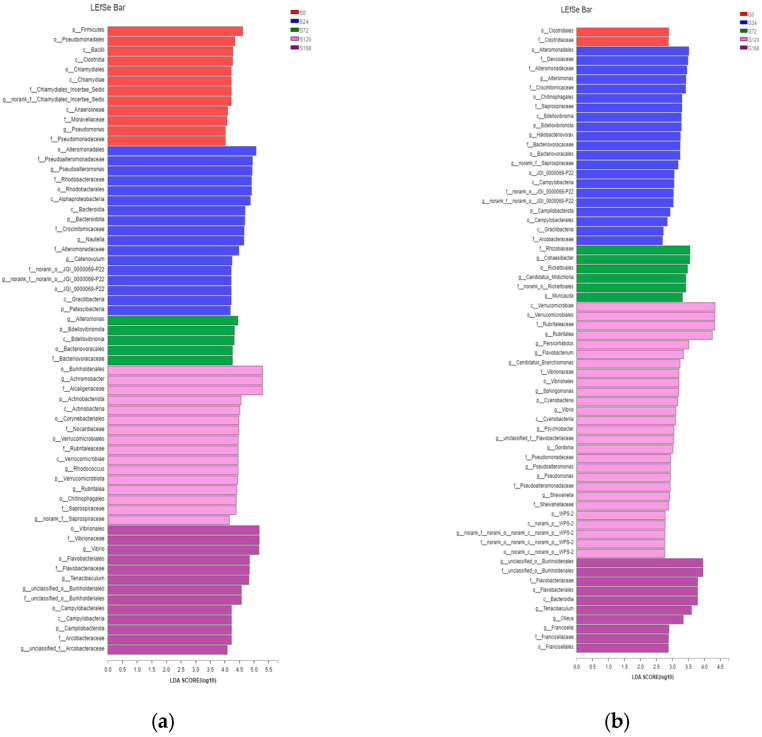
Comparative taxonomic analysis of bacterial community composition across treatments via LEfSe. (**a**) Skin; (**b**) Gill.

LEfSe analysis identified significant differences in characteristic bacterial communities at the phylum and genus levels in skin samples. Specifically, in the S0 group, Firmicutes was the characteristic phylum, while *Pseudomonas* was the characteristic genus. In the S24 group, characteristic phyla included Bacteroidetes and Patescibacteria, with *Pseudoalteromonas* and *Nautella* as characteristic genera. In the S72 group, BdelloVibrionota was the characteristic phylum, with *Alteromonas* as the characteristic genus. In the S120 group, Actinobacteria and Verrucomicrobiota were characteristic phyla, with *Achromobacter* and *Rhodococcus* as characteristic genera. Finally, in the S168 group, Campylobacterota was the characteristic phylum, while *Vibrio* and *Tenacibaculum* were the dominant characteristic genera (Figure 7a).

In gill samples, the G0 group had no characteristic phylum or genus. In the G24 group, characteristic phyla were BdelloVibrionota and Campylobacterota, with *Alteromonas* and *Halobacteriovorax* as characteristic genera. The G72 group had no characteristic phylum, but *Cohaesibacter* was the characteristic genus. The G120 group had Cyanobacteria as a characteristic phylum, with *Flavobacterium*, *Sphingomonas*, *Vibrio*, *Psychrobacter*, *Gordonia*, *Pseudoalteromonas*, *Pseudomonas*, and *Shewanella* as characteristic genera. The G168 group had no characteristic phylum but displayed characteristic genera *Tenacibaculum* and *Francisella* (Figure 7b).

## 4. Discussion

*C. irritans* is a typical marine parasite that can infect most marine teleost fish. Due to its rapid reproduction and spread, outbreaks can cause up to 85% mortality in farmed fish, leading to significant economic losses in the marine aquaculture industry [1,22]. *C. irritans* primarily infects the skin and gills of fish. However, to date, no studies have explored the relationship between *C. irritans* infection and changes in the microbiota of fish skin and gills. Although *C. irritans* can infect nearly all species of marine fish, the degree of harm it causes varies significantly among different host species [23]. The specific characteristics of a fish’s skin and gills influence its tolerance to *C. irritans*. For example, previous studies have demonstrated that the mucus of *Siganus oramin* (yellowspot rabbitfish) contains anti-parasitic proteins with inhibitory or bactericidal effects against both *C. irritans* and various pathogenic bacteria. Consequently, *S. oramin* exhibits innate resistance to *C. irritans*, and even when infected, it rarely induces secondary infections in the host fish [24,25]. Consistently, in the present study, the Simpson index of the G24 group in the gill microbiota was also significantly lower than that of the G0 group. There are relatively few studies on the microbiota of fish gills, and these studies are mainly focused on the relationships between fish gill microbiota and factors such as the aquatic environment, viral infections, and parasitic infections [26,27]. Only one study has reported the effects of *C. irritans* on fish gill microbiota: Xie et al. [28] investigated the impact of *C. irritans* infection on the gill microbiota of the large yellow croaker (*Larimichthys crocea*) and found that infection resulted in substantial microbial changes in the gill. Specifically, the order Rubritaleaceae increased in abundance, while the families Colwelliaceae, Nocardiaceae, and Microbacteriaceae decreased after infection. Additionally, the genera *Ahrensia*, *Cobetia*, *efluviicoccus*, *Rubrivirga*, *Microbacterium*, and *Rhodococcus* disappeared from the gill following infection, whereas *Phaeobacter*, *Peredibacter*, *Aquabacterium*, *Blastocatella*, *Lactococcus*, and *Phycisphaeraceae* SM1A02 attached to the mucosal tissue post-infection. In contrast, in our study, no significant changes in the gill microbiota were observed after infection. These differences may stem from species variations or discrepancies in gill sampling methods. Xie et al. sampled gill tissue, whereas we only collected gill mucus. Since gill tissue is rich in blood, their results might have reflected changes in the blood microbiota within the gills [28].

The symbiotic microbiota plays an indispensable role in mediating host-parasite interactions. Protozoan infections are often accompanied by disruptions in the stability and functionality of host microbiota [9]. Therefore, in research, it is essential to consider the interactions among the host, parasites, and microbiota comprehensively rather than limiting to a host–parasite binary relationship [29,30]. To further reveal the mechanisms of disease caused by parasite infections in fish under natural conditions, research perspectives need to be expanded to cover the more complex host–parasite–microbiota interaction network. Studies have shown that in ecologically balanced networks, interactions between different biological communities play a critical role in maintaining the stability of the microbiota structure [31]. The skin microbiota is a crucial biological barrier against pathogen invasion [32,33,34]. However, parasite infections can disrupt the balance of skin microbiota, increasing the risk of secondary bacterial infections [35,36] and possibly acting as vectors for bacterial pathogens [37]. In Denk’s experiments, they found that different fish species have different propensities to develop lesions when infected with cryptokaryotes. For example, in Chaetodontidae, Lutjanidae, and Kyphosidae, gill lesions are more pronounced, while cutaneous lesions are more prominent in Pomacanthidae. The main causes of the lesions were epidermal hyperplasia and fusion with protozoa. In addition, secondary bacterial dermatitis was found in bigeye bream [38]. Previous studies have demonstrated that during the parasitism of large yellow croaker (*Larimichthys crocea*) by *C. irritans*, the rotatory movement of the parasite on host epithelial tissues induces mechanical damage, leading to the formation of open wounds that serve as direct entry points for waterborne pathogenic bacteria [39]. During mild infections, the host’s immune system can resist bacterial invasion through innate immune responses; however, in cases of severe infection, massive bacterial infiltration occurs due to compromised cutaneous barriers, ultimately triggering secondary bacterial infections. This pathological process is directly associated with the disruption of the cutaneous physical barrier [39]. At the mucosal immune defense level, *C. irritans* infection causes structural damage to epidermal secretory cells, thereby reducing the bioactivity of antimicrobial components (e.g., L-amino acid oxidase) in host mucus. For instance, the natural resistance of rabbitfish (*Siganus oramin*) to parasitic infection is closely linked to potent bactericidal proteins in its mucus and serum [25]. Histopathological observations reveal that when *C. irritans* invades gill tissues, mechanical stimulation and parasitic metabolites induce excessive proliferation of gill mucosal cells, leading to over-secretion of viscous mucus. This pathological response results in adhesion and fusion of gill filaments, severely obstructing water circulation and gas exchange across the gill surface. Consequently, the impaired excretion of metabolic waste and compromised respiratory function culminate in asphyxiation-induced mortality of the host [3]. Collectively, these findings elucidate the pathogenic mechanisms by which *C. irritans* infection causes secondary bacterial infection and host death, encompassing tissue damage, mucosal immune suppression, and respiratory dysfunction.

The skin is the first barrier separating organisms from the external environment, and the mucus layer plays a crucial role in maintaining fish health by providing a physical and chemical barrier between the animal and its surroundings, supporting a highly diverse symbiotic microbial community on different skin sites [40,41]. Several studies have shown that mechanical injuries to fish skin affect the composition of the skin microbiota. For instance, the richness and diversity of the skin microbiota were significantly reduced and the abundance of disease-related microbes such as *Acidovorax*, *Rhizobiaceae*, *Aurantimicrobium,* and *Leifsonia* significantly increased in marble goby (*Oxyeleotris marmoratus*) and grass carp (*Ctenopharyngodon idella*) following mechanical injuries [42,43]. *C. irritans* infections can also cause damage to fish skin. This study found that the changes in the fish skin microbiota caused by *C. irritans* infection were similar to the effects of mechanical injury: as the infection duration increased, the richness and diversity of microbial communities on the skin decreased significantly, reaching their lowest values after *C. irritans* completely detached (Figure 3a). This indicates that *C. irritans* infection not only disrupts the skin integrity of *T. ovatus* but also significantly reduces the diversity and richness of its skin microbiota, disturbing the original microbial community structure and might potentially increase the risk of secondary bacterial infections. Similar to our study, in sea lice (*Caligus rogercresseyi*)-infected Atlantic salmon, the microbiota richness in infected individuals was significantly reduced compared to controls, and the community composition became highly unstable [10]. Additionally, this study has documented that *C. irritans* infection causes skin barrier disruption, thereby providing a route for pathogen invasion; however, it did not explicitly clarify whether mechanical damage acts as the initial trigger for infection. Notably, *C. irritans* infection may indeed promote the success of its own parasitic process by inducing mechanical damage, which could further lead to secondary infections. Therefore, subsequent experiments could be designed to investigate the effects of mechanical versus non-mechanical damage, the degree of parasite infection, and changes in the body surface microbiota.

This study revealed that, at the phylum level, the surface bacterial communities of golden pompano at different stages of *C. irritans* infection were dominated by Proteobacteria, Bacteroidetes, Actinobacteria, and Firmicutes, with Proteobacteria being the most prevalent (Figure 5A). This is consistent with the composition of surface bacterial communities at the phylum level found in other fish species, such as the marble goby (*Oxyeleotris marmoratus*) and grass carp (*Ctenopharyngodon idella*) [42,44].

Within the Proteobacteria phylum, there is a wide variety of bacterial species, and a significant number of these species have been shown to be pathogenic. Studies have demonstrated that changes in the abundance of Proteobacteria may be associated with microbial community instability, dysbiosis of the bacterial community structure, and disease occurrence [45]. Additionally, some research indicates that changes in the abundance of Proteobacteria are related to dysbiosis of the bacterial community structure and the occurrence of diseases in the host [46,47,48]. In our study, compared to the uninfected group, the abundance of Proteobacteria in the surface bacterial communities of samples at each stage after *C. irritans* infection significantly increased, and the abundance of Proteobacteria was positively correlated with the duration of infection, with the highest relative abundance occurring when the parasite had completely shed (Figure 5). This suggests that the surface bacterial community structure is severely imbalanced after the complete shedding of *C. irritans*. To our best knowledge, there has been no report on the changes in Proteobacteria due to parasite infection. Similar to our results, infection with *C. irritans* also significantly altered the intestinal bacterial community structure of the *Epinephelus coioides*, leading to dysbiosis of the intestinal microbiota; however, interestingly, infection with *C. irritans* caused a significant enrichment of Proteobacteria in the intestines of grouper, which is inconsistent with the significant decrease in the abundance of Proteobacteria on the skin of *T. ovatus* after *C. irritans* infection [49]. The reason for the inconsistency may be due to the different species of experimental fish, or it may be that the changes in bacterial community structure caused by *C. irritans* infection are tissue specific.

Most of the genera under the phylum Firmicutes are generally considered beneficial, with prebiotic functions such as maintaining the skin’s acidic environment [50]. Our study also showed that the abundance of Firmicutes in the skin microbiota decreased with prolonged infection (Figure 5A), suggesting a reduction in beneficial bacteria. While studies on changes in fish skin microbiota following parasite infections are limited, research on other aquatic animals experiencing skin damage or stress due to toxicants shows similar trends of reduced Firmicutes abundance. For example, Firmicutes abundance was significantly lower in scratched marble goby (*Oxyeleotris marmoratus*) compared to controls [42]. Povidone-iodine exposure was found to alter the immune response and microbiota of koi carp (*Cyprinus carpio*) gills and skin [51]. Studies have also shown a negative correlation between Firmicutes abundance and inflammation, with Firmicutes abundance increasing after biological treatment for inflammatory diseases, suggesting alleviation of inflammation [52]. Therefore, the decrease in Firmicutes abundance in *T. ovatus* after *C. irritans* infection may also be associated with increased inflammation.

Bacteria of the genus Vibrio are mostly conditionally pathogenic bacteria present in the commensal flora of healthy fish [53]. Previous studies have shown that *Vibrio ponticus* and *Vibrio harveyi* of the genera Vibrio are the main secondary pathogens in the secondary infection of pompano afflicted with the disease caused by the parasite *C. irritans* [54]. Certain Vibrio species have been recognized as both symbiotic components of the normal microbiota in fish and as intracellular symbionts of *C. irritans* [55]. The findings of this study reveal that Vibrio species can be detected in samples from fish not infected with *C. irritans*; however, post-infection, there is a notable upward trend in the relative abundance of Vibrio. Furthermore, as the duration of infection and parasitic persistence lengthens, the relative abundance of *Vibrio* continues to escalate (Figure 7). Comparable to our results, research on the microbiome of Atlantic salmon infested with the sea louse *Caligus rogercresseyi* has shown that *Vibrio* species are predominant within the gut microbiota of the infected hosts [56]. In our study, the relative abundance of Vibrio significantly increased following the onset of parasitism by *C. irritans*. This outcome is likely attributable to heightened stress from the interaction between symbiotic microorganisms and fish, induced by the external stimulus of *C. irritans* infection, a reduction in immunocompetence, and diminished resistance to infection, allowing the proliferation of opportunistic pathogens such as *Vibrio*.

Bacteria of the genus *Nautella* are commonly found in marine settings and have been shown to be pathogenic to the red alga *Delisea pulchra* [57]. They are also known to be symbiotic with rotifers [58]. Their abundance was also reported to increase when the host was in an unfavorable physical condition. For example, when conducting pathogen isolation from diseased *Litopenaeus vannamei*, it was observed that *Nautella* is the second most prevalent conditional pathogen following the Vibrio genus. Research on the toxicity of titanium dioxide nanoparticles to grouper fish revealed that the relative abundance of *Nautella* in the intestines of fish exposed to these nanoparticles was significantly higher than in the control group (*p* < 0.05) [59]. In our study, we found that *Nautella* was undetectable in the mucous samples from the skin of uninfected *T. ovatus*, but its abundance peaked 24 h post-infection with *C. irritans* (Figure 6A). As the parasite matured and eventually shed, the abundance of *Nautella* decreased. We speculate that this fluctuation in *Nautella* abundance could be attributed to the following: (1) the parasite’s invasion damaging the fish’s surface, allowing pathogens from the *Nautella* to appear on the surface; (2) the stress from the parasite infection leading to a weakened immune system, causing a surge in the conditional pathogen during the early stages of infection; (3) as the host adjusts to the stress of the infection, its resistance to the pathogen increases, resulting in a reduction in *Nautella*; and (4) the parasite’s detachment from the host, taking *Nautella* bacteria with it. However, since the experiment did not assess the characteristics and quantity of *Nautella* bacteria in the water environment and those carried by *C. irritans*, these speculations need further validation through additional experiments.

The bacteria of the genus *Achromobacter* are opportunistic pathogens, mainly existing in moist environments. Since 2017, they have been primarily isolated from the respiratory tracts of patients with cystic fibrosis (CF). In recent years, they have attracted significant attention due to their frequent isolation from patients with pneumonia [60]. Human medical research has found that *Achromobacter* infections typically occur in patients with underlying immune deficiencies. The abundance of these bacteria is usually positively correlated with the severity of the host’s infection [61]. The bacteria of the genus *Rhodococcus* are also opportunistic pathogens widely found in the environment. They were first isolated as pathogens from a pony with pneumonia in 1923. The first human case was reported in 1967. Studies have found that most infections caused by *Rhodococcus* occur in hosts with compromised immune function, most typically those with cellular-mediated immune deficiencies [61]. The abundance of *Rhodococcus* is also usually associated with the host’s inflammatory diseases [62]. Our research found that the abundance changes in both *Achromobacter* and *Rhodococcus* genera show similar trends before and after infection: the uninfected group had the lowest values, and the abundance gradually increased with the duration of infection, peaking at the end of the infection (120 h), and significantly decreasing after the parasites fell off. It is speculated that the changes in the abundance of these two genera are mainly related to the host’s inflammatory condition (Figure 6A). When the *T. ovatus* is stimulated by infection with the parasite *C. irritans* and inflammation occurs on the skin, the abundance of these two genera on the skin also increases. After the parasites fall off, as the skin integrity is restored, the abundance of these two genera also decreases. However, since this experiment did not detect changes in host inflammatory-related indicators, the above speculations still need further experimental verification.

Our study of the abundance of pathogenic microbial flora revealed that, after infection with *C irritans*, the total abundance of pathogenic microorganisms increased sharply from 7.66% in the uninfected group to 53.97% in the infected group and remained at a high level. It is evident that *C irritans* infection increases the susceptibility of *T. ovatus* to bacterial pathogens, which is similar to the findings of Bandilla [63] in their study on rainbow trout: in vitro parasitic infection increases the susceptibility of rainbow trout to bacterial pathogens such as *Vibrio*. This increased susceptibility could be direct, for example, when parasitic-induced skin damage creates an entry point for bacteria [35,36] or when the parasite acts as a vector for disease (Cusack and Cone, 1986) [37]. The increased susceptibility could also be indirect, such as mechanically stimulating the skin mucus of grass carp (*Ctenopharyngodon idella*), resulting in a significant change in the activity of immune-related enzymes like lysozyme in the skin mucus, thereby altering the composition of the microbial community in the skin mucus, disrupting the balance between the host and symbiotic bacteria, increasing conditional pathogenic bacteria, and reducing beneficial bacteria, thus leading to increased susceptibility of fish to bacterial pathogens [64]. Furthermore, our study indicates that with the increase in the duration of *C*. *irritans* infection, the bacterial load in the liver of *T. ovatus* also significantly increases, reaching its highest value during the parasite shedding phase (Figure 1). It is clear that the degree of secondary infection in the body of *T. ovatus* progresses with the advancement of parasitic infection. In a related study on large yellow croaker infected with *C. irritans*, it was shown that the bacteria within the large yellow croaker were secondary infections, which were the pathogenic bacteria causing the mass mortality of the fish, and the infection pathway was water → wound → body [39]. It is speculated that the pathway by which *C. irritans* infection leads to secondary infection in the liver of *T. ovatus* in this study is as follows: as the infection stage of *C. irritans* progresses, the balance of the surface symbiotic bacterial community is disrupted, increasing conditional pathogenic bacteria and reducing beneficial bacteria. Secondary bacterial infection requires adhesion and colonization and the destruction of the first line of defense, which may be achieved by the destruction of the skin mucus and skin, the first line of defense, during the infection and shedding process of *C. irritans*, thus opening the channel for conditional pathogenic bacteria to invade. At the same time, the infection of *C. irritans* leads to a decrease in host immunity, weakening the host’s control over conditional pathogenic bacteria, and thus, with the infection of *C. irritans*, opportunistic pathogenic bacteria take advantage of the situation and proliferate massively within the fish, thereby causing secondary infection of the liver. However, whether the secondary infection caused by *C. irritans* infection in *T. ovatus* is a direct or indirect factor, or both, this study cannot directly determine. Further experiments need to be designed for verification.

This study primarily employed 16S rRNA amplicon sequencing, a widely used method for analyzing microbial community composition. However, it has several inherent limitations. The taxonomic resolution is relatively low, making it difficult to distinguish closely related species. Primer bias during amplification may result in the underrepresentation or omission of certain microbial groups. Moreover, this method provides only taxonomic profiles without offering insights into functional genes or microbial activity. It also cannot detect non-bacterial microorganisms, has limited quantitative accuracy, and is susceptible to contamination and chimera formation during PCR. Therefore, for studies requiring higher taxonomic resolution or functional characterization, it is recommended to complement 16S sequencing with metagenomic approaches or full-length 16S sequencing technologies. Additionally, this method is only capable of revealing the taxonomic composition of microorganisms and cannot confirm whether the identified Vibrio strains possess pathogenic potential. Future studies can utilize PCR amplification of virulence-associated genes (e.g., *toxR*, *tdh*, and *trh*) or metagenomic analysis to validate the pathogenicity of Vibrio strains.

## 5. Conclusions

This study conducts an in-depth analysis of the impact of *C. irritans* infection on the composition of the skin microbiota in *T. ovatus*. The results indicate a notable decrease in the species richness and diversity of the skin microbiota following infection with *C. irritans*. A subsequent increase in microbial richness is observed after the pathogen detaches from the host. A detailed examination of the microbiota composition reveals that, at the phylum level, Proteobacteria remain dominant throughout the infection period, whereas the abundance of Firmicutes decreases as the duration of infection increases. At the genus level, there is a significant increase in the relative abundance of opportunistic pathogenic genera, including *Vibrio*, *Nautella*, *Pseudomonas*, and *Rhodococcus*, during *C. irritans* infection. However, no significant shifts were observed in the gill microbiota following *C. irritans* infection. The dominant phyla and genera of the gill microbiota remained largely consistent, with no substantial alterations, as the parasite infection period progressed.

This study delves into the effects of parasitic infection on the equilibrium of the host’s microbiota and examines the potential mechanisms through which *C. irritans* infection may lead to secondary infections in *T. ovatus*. The findings offer novel scientific insights and guidance for the development of prevention and control measures against parasitic infections, particularly for parasites such as *C. irritans*.

## Data Availability

The original contributions presented in this study are included in the article. Further inquiries can be directed to the corresponding authors.
